# Towards a molecular basis of oligometastatic disease: potential role of micro-RNAs

**DOI:** 10.1007/s10585-014-9664-3

**Published:** 2014-06-27

**Authors:** Abhineet Uppal, Mark K. Ferguson, Mitchell C. Posner, Samuel Hellman, Nikolai N. Khodarev, Ralph R. Weichselbaum

**Affiliations:** 1 Department of Surgery, The University of Chicago, MC 5029, 5841 S. Maryland Ave, Chicago, IL 60637 USA; 2 Department of Radiation and Cellular Oncology, Ludwig Center for Metastasis Research, The University of Chicago, 900 East 57th Street, Chicago, IL 60637 USA

**Keywords:** Oligometastases, Metastases, Micro-RNA, Breast cancer, Lung cancer, Colorectal cancer

## Abstract

Oligometastasis is a cancer disease state characterized by a limited number of metastatic tumors involving single or few organs and with biological properties that make them potentially amenable to locoregional antitumor therapy. Current clinical data show that they are potentially curable with surgical resection or/and radiotherapy. Yet, mechanisms of progression from primary tumor to oligometastasis, rather than to polymetastases, is lacking in detail. In the current review we focus on the role of micro-RNAs in the regulation of metastases development and the role they may play in the differentiation of oligometastatic from polymetastatic progression. We also discuss the analyses of metastatic samples from oligo-and polymetastatic patients, which suggest that oligometastasis is a distinct biologic entity regulated in part by micro-RNAs. In addition, a review of the known functions of oligometastatic-specific micro-RNAs suggest that they regulate multiple steps in the metastatic cascade, including epithelial–mesenchymal transition, tumor invasion, intravasation, distant vascular extravasation and proliferation in a distant organ. Understanding the role of micro-RNAs and their target genes in oligometastatic disease may allow for the development of targeted therapies to effectively conrol the spread of metastases.

## Introduction

Current approaches to the staging of cancer patients are based on the separation of primary or locoregional disease and distant metastases. Such separation depends on the ability to apply curative surgical or radiotherapy approaches to the primary tumors or locoregional disease, while distant metastases are commonly treated by palliative systemic therapy [1, 2]. The heterogeneity of metastatic spread is generally underestimated and many clinical regimens are not based on the extent of metastatic dissemination. The term “oligometastases” was introduced in 1995 and describes an intermediate state of metastatic spread between localized disease and diffuse metastases [3]. The initial definition of oligometastases was introduced mostly based on the limited number of secondary lesions, but more recently, the rate of metastases development was taken in consideration with introduction of the concept of “oligorecurrence” [4–6]. The concept of oligometastatic disease has significant clinical value because a limited number of lesions can be treated with the same potentially ablative approaches as spatially confined primary tumors. Previously, the feasibility of this approach was demonstrated mostly with surgical intervention in limited number of cancers [7, 8]. More recently, rapid advances in imaging techniques and stereotactic body radiotherapy (SBRT) allow earlier detection of oligometastases and non-invasive or minimally invasive intervention in cancers with different histologies and different secondary sites [9–11]. Results from large-scale randomized controlled studies are not available, but recent comprehensive reviews of the literature suggest that approximately 20% of patients may represent an oligometastatic phenotype where local treatment of metastases by resection, ablation or radiotherapy yields outcomes that are substantially improved when compared to non-treated patients [12–15]. Yet, little is known so far about the molecular basis of oligometastases and the pathways that can be responsible for differentiating between polymetastatic or oligometastatic spread.

In the current report we discuss the potential role of micro-RNAs in oligometastatic disease based on known examples of regulation of the metastatic cascade by micro-RNAs, along with our initial investigations of micro-RNA expression in the secondary lesions of oligometastatic and polymetastatic patients.

## Oligometastases versus polymetastases: are there genome-driven mechanisms?

**Fig. 1:**
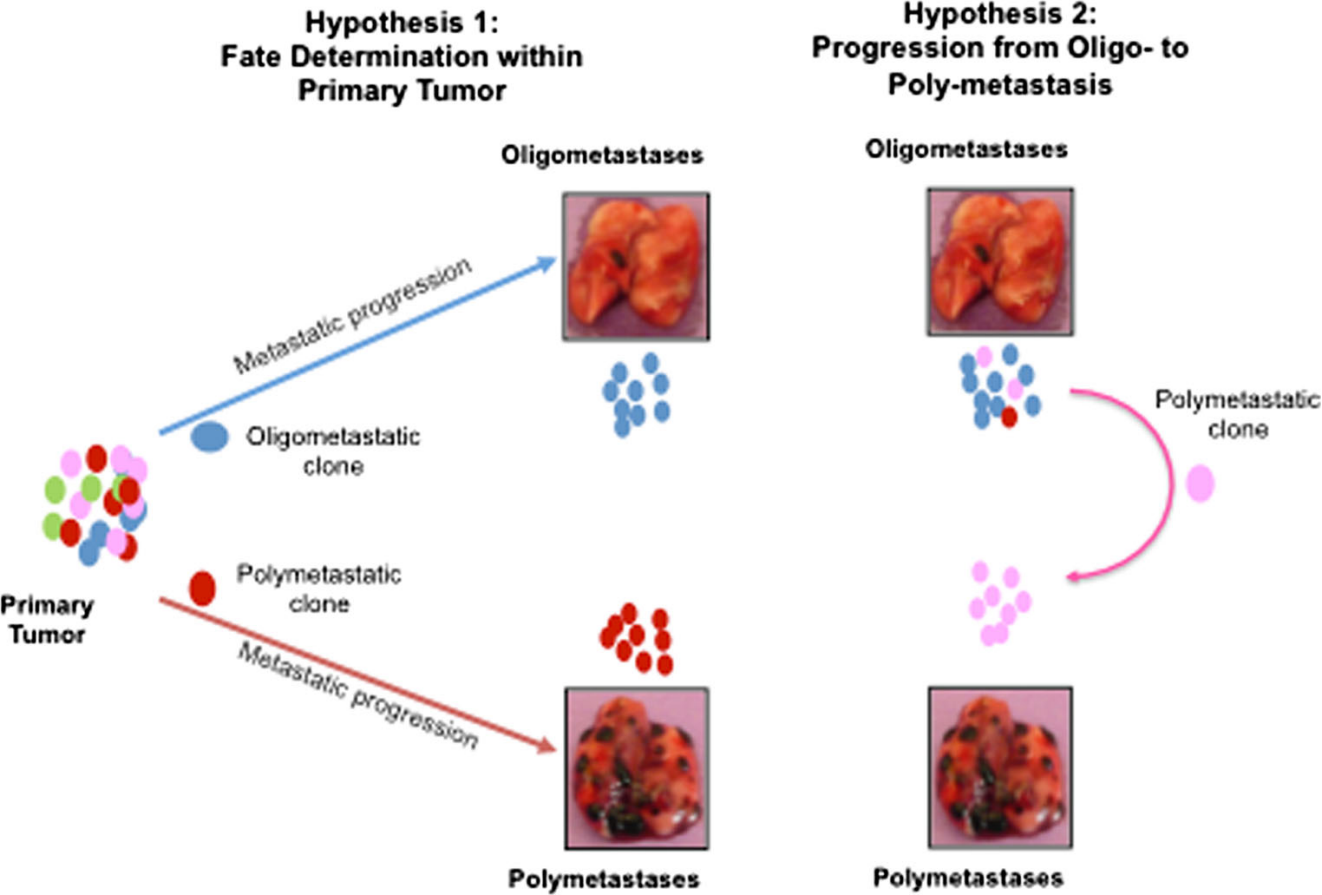
Pathways of oligo-and polymetastases development. Two hypotheses of Oligometastastic Disease: *Hypothesis 1* Oligometastases and Polymetastases may be distinct metastasis phenotypes determined by dissemination of clonal populations with differing metastatic potential. *Hypothesis 2* Metastasis may be a continuum of phenotypes identified early (oligometastases) or late (polymetastases) in the progression of disease

Despite advances in the understanding of the clinical significance of oligometastasis, comparative molecular and genomic investigations of oligometastatic and polymetastatic lesions are limited. Two extreme possibilities implicate either a clonal origin with inherited molecular and phenotypic differences of oligo-or polymetastases, or sequential development of the metastatic process with oligometastases as an early but transient phase of polymetastatic disease (Fig. 1). Current data based on genomics approaches are consistent with both suggestions. The clonal nature of metastases and their origin from topographically and genetically distinct regions of tumors has been demonstrated in the next-generation sequencing of the primary and secondary tumor samples taken from the same patients [16–18]. Distinct geographic regions of primary pancreatic tumors represented different clonal structure and gave origin to the distinct metastatic clones [16, 17]. In clear-cell renal carcinoma it was demonstrated that metastatic clones could share common mutational events with ancestor clones in the primary tumor [19]. In breast cancer, application of single-cell sequencing approaches revealed a clonal sub-structure of primary tumors and the initiation of liver metastases as the result of expansion of individual primary clones [18, 20]. These studies demonstrated that secondary tumors are derived from individual clones of heterogeneous primary tumors and follow the rules of Darwinian evolution [21, 22]. Therefore it is reasonable to suggest that secondary lesions with an oligometastatic phenotype may originate from the tumor clones with genetic properties different from clones potentially “programmed” towards polymetastatic dissemination. Detection of these differences can be pivotal in identifying prognostic/predictive markers of metastatic disease and detection of new therapy targets. In addition, constant evolution and “re-seeding” have been demonstrated [17, 23]. This raises the possibility that oligometastases may be a transient stage of general metastatic progression (Fig. 1). The time scale of spontaneous metastases development, estimated from genomic differences between matched primary and secondary tumors, varies between 1 year to 5 decades [24], providing a variety of opportunities for the evolution from restricted oligometastatic clones to disseminated disease either through tertiary metastases or progressive evolution of the primary tumor [17]. Genomic and transcriptomic profiling of oligo-and polymetastatic lesions may reveal recurrent mutations/patterns common for both types of secondary tumors or identify distinguishing characteristics that separate biologic processes responsible for distinct phenotypes.

## Multi-step nature of metastatic cascades as the basis for differences in oligo-and polymetastatic progression

**Fig. 2:**
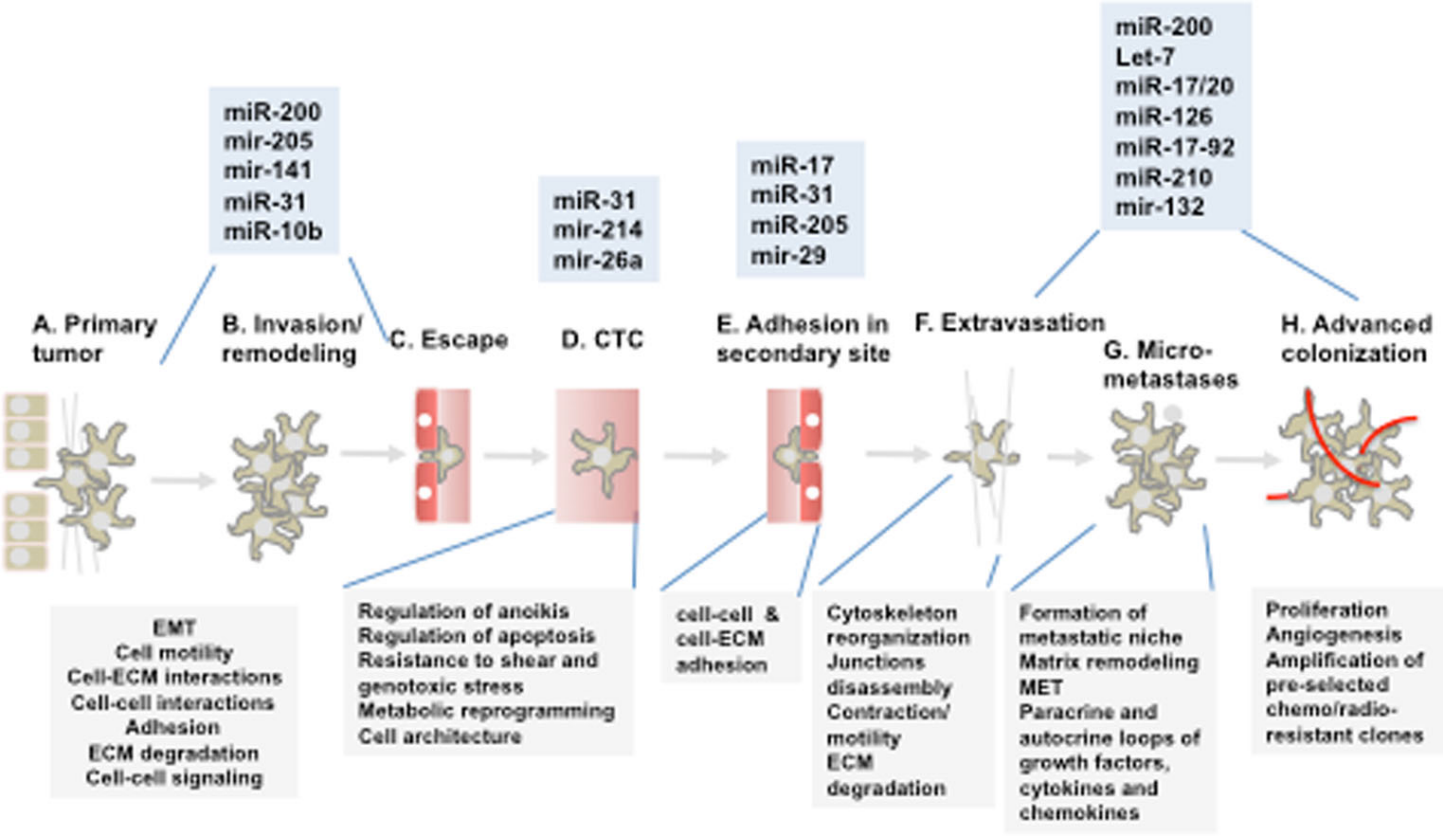
Multiple steps of metastases development and role of micro-RNA in their regulation. Sequential steps of metastases development are presented based on the current reviews [25, 26, 28, 51]. Note that final selection of metastatic clones can lead to the formation of radio/chemoresistant secondary tumors, as was described elsewhere [33], presumably due to selection against cytotoxic factors of host microenvironment (see *g*–*h*). *Boxes* below diagram represent major functions involved in each step, while *boxes* above illustrate some micro-RNAs involved in the regulation of each step (see text for explanations and references)

Based on advances of high-throughput genomic, transcriptomic and proteomic approaches, the last decade has brought important breakthroughs in our understanding of the metastasis process. It is commonly accepted now that metastases formation is a multi-step process with defined steps (Fig. 2). Understanding of the specific biological processes and pathways underlying each step is rapidly evolving (reviewed in [25–28]). This multi-step nature of metastatic cascades suggests that divergence between oligometastases and polymetastases may be initiated on different steps of metastases development. Each step can provide a bifurcation between the evolutionary pathways of these metastatic types, though it is possible to speculate that bifurcations in the earlier steps (such as invasion/remodeling, Fig 2b) can lead to more pronounced differences between oligo-and polymetastatic phenotypes as compared with bifurcations at the later steps (Fig. 2g, h). This multi-step nature of metastatic cascades can induce heterogeneity even within these two metastatic phenotypes. Indeed, recently Tree et al. [12] noticed that at least three different cohorts of patients were reported as oligometastatic: those who present at time of diagnosis with oligometastatic disease (“synchronous” oligometastases), those who had truly oligometastatic relapse after curative locoregional therapy of primary disease (metachronous oligometastases) and those with “induced” oligometastases after cytoreductive therapy. These groups probably have different prognoses and benefit from different therapeutic approaches. Detailed investigations of these steps in the context of oligometastases and polymetastases development are necessary for detection of prognostic markers and potential therapeutic targets with potential clinical applications.

Examination of biological processes implicated in the different stages of metastases development leads to an important conclusion: if the most prominent hallmark of cancer growth—sustained proliferation [29]—is critical for the development of primary tumors and advanced colonization (Fig. 2a, h), all intermediate steps of metastases development are based on the processes not directly related to the regulation of tumor growth. Rather, they involve multiple interactions of metastatic cells with microenvironment either through direct heterotypic cell–cell and cell–ECM contacts (as in steps 2b–2f) or formation of multiple paracrine and autocrine loops between tumor and stromal cells via secreted ligands and their cognate receptors (Fig. 2g) [30, 31, 32]. Of note, formation of these auto-paracrine loops is often associated with development of tumor clones resistant to genotoxic stress [33–36]. These processes involve constant alterations of the cytoskeleton and cell architecture associated with motility, invasion and circulation in blood. This suggests that metastases development might be regulated through mechanisms different from traditional oncogenic signaling, and instead adopted to the tumor plasticity typical for metastatic cascades. Accumulating evidences indicate that one of the most important mechanisms of regulation of the broad set of pathways involved in metastases regulation is based on micro-RNAs.

## Micro-RNAs as systemic regulators of metastases development

**Fig. 3:**
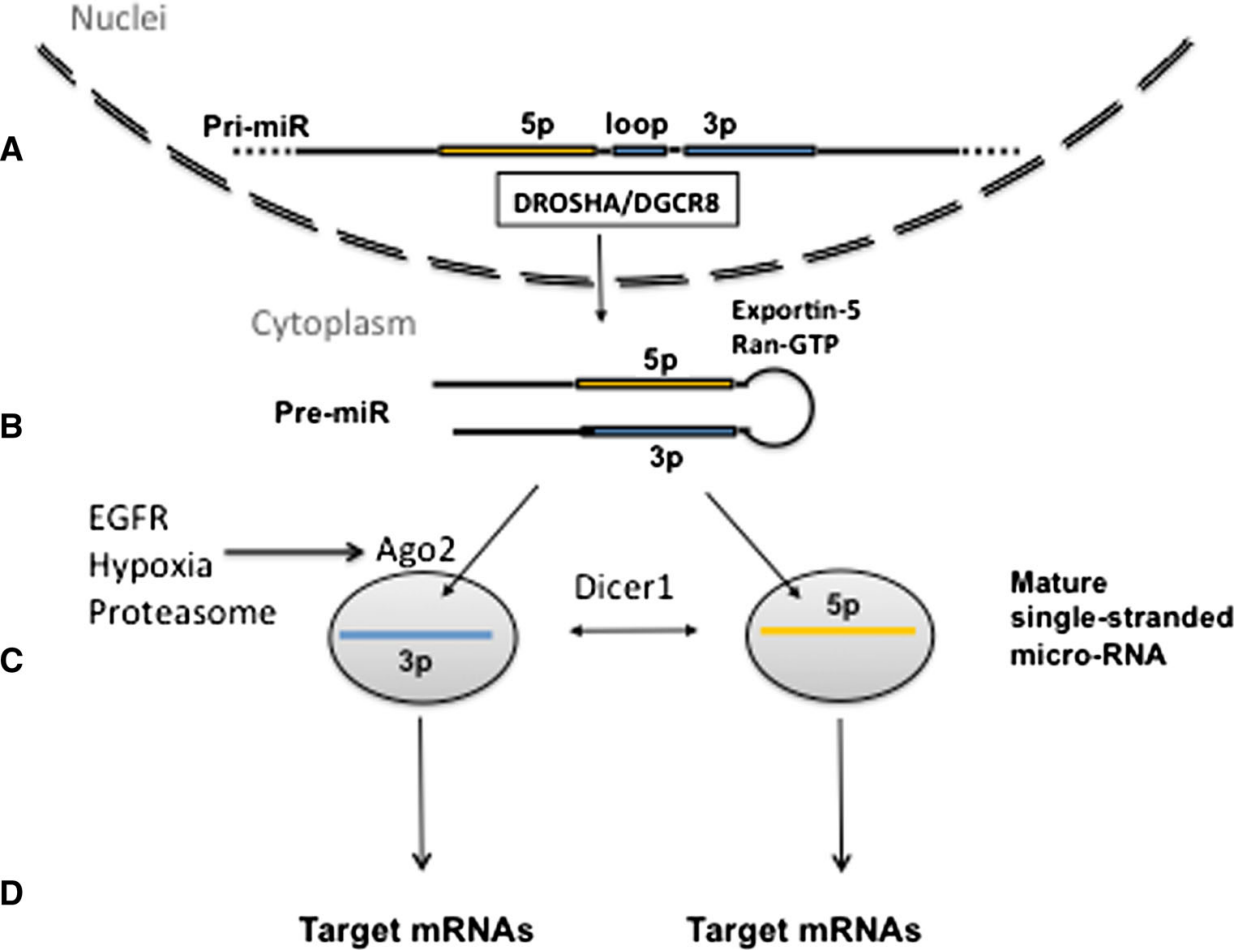
Processing and maturation of micro-RNAs. Genomic sequence of primary micro-RNA (pri-miR) encodes ~80 nt sequence of the stem-loop pre-micro-RNA (Fig. 3a). This stem-loop structure (pre-miR) is recognized and excised from transcribed RNA by multiple proteins including Drosha and DGCR8. The stem-loop pre-miR is exported in cytoplasm by Exportin-5/Ran-GTP complexes (Fig. 3b) and is further processed by Ago2/Dicer1 nucleases into the mature single-stranded 5p and 3p miRs (Fig. 3c). The 5p and 3p mature single-stranded miRs will bind to different down-stream target mRNAs and can lead to different functional outcomes (Fig. 3d). Therefore the balance of micro-RNA precursors and mature forms can determine different phenotypes of normal and tumor cells. Current data indicate that this balance is susceptible to post-translational modifications and/or mutations of enzymes, responsible for miRs processing, including Ago2 and Dicer1 (Fig. 3c). These mutations and post-translational modifications of Ago2, associated with EGFR signaling, hypoxia and proteasome functions are described elsewhere [18–20]

MicroRNAs (miRs) are small (19–22 nucleotide) RNAs involved in the regulation of gene expression. A full description of the biogenesis and complex functions of miRs is beyond the scope of this review and is available elsewhere [37–39]. Briefly, precursors of miRs are encoded by intronic regions of protein encoding genes or by RNA encoding regions of the genome known as primary microRNAs (pri-miRs). They are processed and translocated into the cytoplasm in the form of double-stranded stem-loop structures known as precursor microRNAs (pre-miRs) with the involvement of multiple nucleases including the Drosha, Dicer and Argonaut families (Fig. 2). Importantly, recent investigations detected systemic changes in micro-RNA processing, with imbalance between pre-miRs and mature miRs, and between 3p and 5p mature miRs, due to Ago2 protein modifications by EGFR and other regulators [40, 41] in addition to tumor-related somatic mutations of Dicer1 [42]. Suppression of Ago2 can lead to the decrease of the ratio of mature miRs to stem-loop precursors, while mutations in Dicer1 are associated with the decrease of 5p to 3p ratio of mature single-stranded miRs (Fig. 3). These observations indicate that functional or mutational changes of single proteins involved in the processing of miRs can affect hundreds of down-stream genes. They also indicate that in the analysis of micro-RNA expression, it is important to account for the balance between pre-miRs and mature miRs and the balance between 5p and 3p mature miRs. Each mature single-stranded miR binds to target mRNAs through complimentary seed sequences 6 to 8 nucleotides in length. Longer mRNA complimentary sites and the presence of adenosine immediately 5’ of the site may increase mRNA suppression (reviewed in [43] ). Upon binding, primarily to the 3’ untranslated region (UTR) of the mRNA, miRs induce either suppression of mRNA translation or degradation of the mRNA itself. Each miR is able to bind to hundreds of target mRNAs, thus they represent a potent group of regulators of gene expression.

Micro-RNAs’ extensive regulatory ability has engendered intensive investigation in the context of metastases development. Micro RNAs can mediate the formation of the flexible networks involved in the regulation of the multistep metastatic cascades. Micro-RNAs have also been shown to be regulated by p53 and myc, leading to either inhibition or activation of pro-survival mechanisms necessary for initial proliferation of the primary tumor [44]. This step can also be modulated by regulation of DNA repair and epigenetic modifications to DNA by micro-RNAs, and the converse regulation of micro-RNAs by these mechanisms, as has been highlighted in cancers with micro-satellite instability [45].

Proceeding along the metastatic cascade, many micro-RNAs have been implicated in regulation of epithelial to mesenchymal transition (EMT). One of the most studied families of micro-RNAs is the miR-200 family, which has been implicated with the maintenance of an epithelial phenotype (preventing EMT) via its gene targets Zeb1 and Zeb2, the transcriptional suppressors of E-cadherin [46, 47, 48]. This suppression prevents cancer cells in primary tumors from initiating the process of metastasis. Mir-205 is also implicated in EMT suppression through a feedback loop between Zeb1 and TGFB1 [49]. A similar function can be performed by mir-203 through formation of negative feedback loop with Snail [50]. It is clear that epithelial cell plasticity plays an important role in metastatic cascades, and the role of other micro-RNAs in this process is under intensive investigations as detailed below.

Concurrent with EMT is invasion of tumor cells through the basement membrane, a process extensively regulated by many micro-RNAs through regulation of Rho-mediated actin cytoskeleton regulation, integrin-mediated cell-cell adhesion and alterations in the extra-cellular matrix itself (reviewed in [51]). Another well-studied example of regulation at this step is miR-31, which suppresses invasion/migration of tumor cells, thereby suppressing the initial stages of metastases [52]. Proceeding along the metastatic cascade, the step of adhesion in the second site is critical for determination of site-specificity of a given tumor. Interestingly, due to the targeting of RhoA and ITGA5, miR-31 is critical in adhesion of circulating tumor cells, therefore providing one of the few known “master oncomirs” [52]. Similarly, miR-214 through suppression of AP2-transcription factor TFAP2C and Integrin alpha-3, can also promote both invasion and survival in anoikis [53]. This step of survival in circulation can also be promoted by miR-26a through Rb1-E2F1 pathway [54]. Some examples of metastases-related miRs are presented in Fig. 2. The final stages of metastases formation include initial colonization and subsequent proliferation in distant sites. These steps involve multiple miRs regulating the mesenchymal-to-epithelial transition (MET), formation of a pre-metastatic niche, adaptation of surviving tumor cells to the new microenvironment, secondary tumor growth and angiogenesis (Fig. 2g, h). Multiple miRs can regulate the phenotype of tumor cells in these stages. The oncogenic miR-17-92 cluster can be up-regulated by c-Myc, E2F and Hedgehog [55, 56] and suppresses thrombospondin 1, thereby promoting angiogenesis [57]. Let-7 and miR-126 can inhibit proliferation through HMGA2 and PIK3 signaling [58, 59], while the hypoxia-inducible miR-210 is involved in mitochondria stabilization and regulation of cell survival/proliferation (reviewed by [60]). An understanding of both the pro- and anti-metastatic effects of micro-RNA regulated networks is leading to an appreciation of the role of both tumor cells and the micro-environment in metastasis (further reviewed in [61]). These data suggest that analysis of miRs may lead to the identification of the biological basis of oligometastases and perhaps differentiate between patients who are truly oligometastatic or those who are at risk for widespread distant disease.

## Detection of micro-RNAs associated with oligometastases development (oligomiRs)

As described above, microRNAs are potent regulators of metastatic cascades, providing flexible regulatory networks for metastases development. Yet, due to the limited number of studies comparing oligometastatic to polymetastatic clinical samples, little is known about their role in oligometastases development. To detect microRNAs associated with oligometastases and discriminate them from polymetastases, we investigated archival samples from two clinical databases of oligometastatic and polymetastatic patients [6, 62]. These investigations focused on the direct comparison of oligo-and polymetastatic secondary lesions rather than on comparison of primary and secondary tumors. This led us to the detection of microRNA patterns specific to oligometastatic lesions and suggested the differential regulation of specific molecular pathways that guide oligometastatic vs. polymetastatic development of secondary tumors. We will further name these oligometastases-specific microRNAs as oligomiRs.

## Micro-RNA signatures of surgically resected lung metastasis with oligo-and polymetastatic progression

Sixty-three patients with available metastatic samples were considered oligometastatic at the time of pulmonary resection, with 5 or less metastases. Based on the analysis of recurrence patterns in the lung following resection, 32 patients were designated as slow progressors, corresponding to the oligometastatic phenotype and 16 were classified as rapid progressors, corresponding to the polymetastatic phenotype. These patients had dramatic differences in survival, as demonstrated by Kaplan–Meier analysis, in favor of the slow progressors [6].

**Fig. 4:**
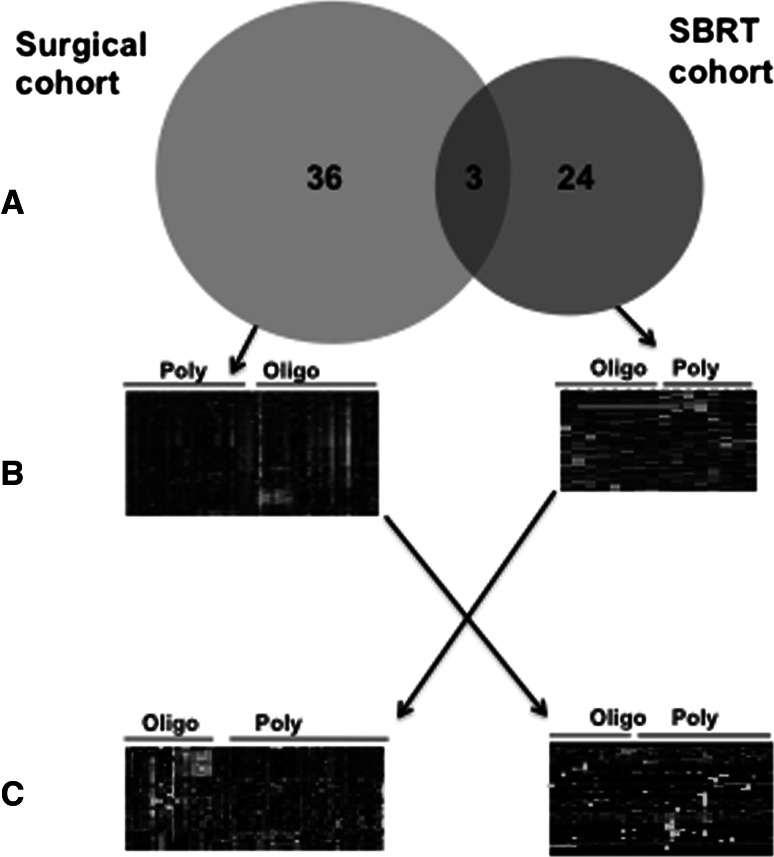
Cross-talk between micro-RNA patterns obtained in surgical and stereotactic body radiotherapy (SBRT) cohorts. **a** Only 3 overlapping micro-RNAs were identified in the surgical and SBRT cohorts (miR-328, miR-502-5p and miR-199b-5p (Table 1). **b** Unsupervised clustering of patients in the surgical and SBRT cohorts based on differentially expressed micro-RNAs successfully segregated patients with oligo-and polymetastatic disease independently on clinical parameters of disease progression (heat maps represent normalized CT values; green are up- and red are down-regulated micro-RNAs). **c** Application of the SBRT micro-RNA signature to surgical patients successfully separated them into oligo-and polymetastatic clusters (*left panel*); the same was true for application of the surgery micro-RNA signature to SBRT cohort

Statistical analysis revealed that 39 micro-RNAs were differentially expressed between oligometastatic (slow progressors) and polymetastatic (rapid progressors) samples. Only three micro-RNAs were down regulated in oligometastases as compared to polymetastases; the remainder were up regulated (Fig. 4b). As described below, several miRNAs up regulated in oligometastases have been characterized as tumor suppressors, implying that their up-regulation may repress oncogenic genes/pathways and limit metastatic spread (Table 1). Therefore these experiments revealed that oligometastases of different primary origin that develop in the same secondary site (lungs) express patterns of tumor-suppressive micro-RNAs, which differentiates them from polymetastases.

## OligomiRs differentially expressed in patients treated with Stereotactic Body Radiotherapy (SBRT)

Stereotactic body radiotherapy is a form of radiotherapy that exploits advances in radiotherapy technology, computing and imaging to deliver high doses of radiation to local tumors. Patients enrolled in a clinical trial, reported by Lussier et al. [14, 63], had diverse primary and metastatic sites, received adjuvant therapy or/and surgery prior to enrollment and had controlled primary tumors with five or less metastases. Patients were classified into two groups based on radiographic response after completion of SBRT: *polymetastatic* patients had (1) progression with development of 5 new metastatic tumors in less than 4 months from time of first metastatic progression, or (2) progression within a body cavity suggesting the presence of diffuse metastatic disease. In contrast, *oligometastatic* patients had either no evidence of progression or experienced progression that did not satisfy the above criteria for designation of polymetastases. The expression patterns of micro-RNAs from metastatic tumors correctly classified seven of nine oligometastatic patients and five of five poly-metastatic patients (Fig. 4b). Twenty-nine differentially expressed oligomiRs were identified in this dataset—16 were down-regulated and 13 up-regulated in oligometastases relative to polymetastases (Table 1). Importantly, in five patients with available matched primary and metastatic tumors, samples of primary and secondary tumors were co-clustered. This suggests that secondary tumors inherit patterns of micro-RNAs expressed in primary tumors (Fig. 1a). On the other hand, analysis of the primary tumors was unable to statistically significantly separate the oligo-and polymetastatic patients based on primary micro-RNA patterns. This lack of discrimination may be due to the heterogeneity of primary tumors, wherein the micro-RNA expression patterns of those clones that can metastasize are masked by numerous non-metastasizing clones (Fig. 1). In genetically more homogenous metastatic tumors, these patterns can be revealed and potentially used for prognostic/predictive purposes. Development of micro-RNA signatures associated with types of metastases progression and/or response to local therapy can lead to improved prognostic tools for oligometastatic patients.

## Cross-talk between oligomiRs patterns in two independent databases

Patterns of oligomiRs detected in each cohort of patients were able to stratify patients in oligo-and polymetastatic phenotype independently from clinical parameters (7, 24). In the surgery cohort, microRNA expression successfully classified 13 of 16 (81.3%) polymetastatic patients and 20 of 32 (62.5%) oligometastatic [odds ratio = 7.22; *p* = 0.006, Fisher’s exact test (FET), 24]. Similarly, in SBRT cohort patterns of oligomiRs successfully classified 8 of 10 (80%) oligometastatic patients and 6 of 6 (100%) polymetastatic (*p* = 0.007, FET, [7]). These separations are schematically depicted in Fig. 4b. At the same time, both patterns did not overlap significantly; only three microRNA were common between oligomiRs from surgery and SBRT cohorts (miR-328, miR-502-5p and miR-199b-5p; see Fig. 4a). Yet, when we used 39 oligomiRs detected in the surgery cohort to classify patients in the SBRT cohort, we obtained significant separation of oligo-and polymetastatic patients (OR = 7.2, *p* = 0.047, FET; Fig. 4c). Similarly, when we applied 27 oligomiRs from the SBRT cohort to classify patients in the surgery cohort we observed divergence of oligo-and polymetastatic phenotypes (OR = 3.9, *p* = 0.032, FET). These findings suggest that both sets of detected oligomiRs are actually co-expressed in oligometastatic lesions from two independent clinical cohorts, but cannot be simultaneously detected due to the limited statistical power of these smaller sample sets. Further studies incorporating larger numbers of samples and more sensitive profiling platforms will lead to extended list of oligomiRs commonly shared between different clinical groups of patients.

## Biological validation of clinical observations-controversial functions of mir-200c in oligometastases development

Detection of oligomiRs differentially expressed in oligometastases as compared with polymetastases posed questions about their functional significance. We found in the SBRT cohort three up-regulated micro-RNAs in polymetastases that were members of the mir-200 family (mir-200b, mir-200c and mir-141). We suggested that if these differentially expressed micro-RNAs are involved in the regulation of metastatic phenotypes, their ectopic expression in oligometastatic tumor may switch its phenotype to polymetastatic. To test this concept two models were used: a syngeneic mouse melanoma B16 model with oligo and poly-metastatic clones derived as described elsewhere [33], and a human MB-435 breast carcinoma model with oligo- and poly-metastatic clones selected in vivo. Over-expression of mir-200c was sufficient to switch the oligometastatic phenotypes to polymetastatic phenotypes in both models. These findings indicated that oligomiRs detected in our clinical datasets may be causal in the regulation of oligo- vs. poly-metastatic development (7). At the same time these were paradoxical observations, since extensive literature indicate that mir-200c is a tumor suppressor (Fig. 2) [26, 27]. However, our data suggested that cells that had escaped the primary tumor were more metastatic when expressing microRNA 200c. Currently these results are supported by those of Dykxhoorn et al. [64] and Korpal et al. [65], both of which demonstrated that expression of 200c increases the metastatic phenotype in tumor cells that escape the primary tumor by enforcing the epithelial phenotype and/or suppressing tumor suppressive proteins induced by Sec23a, a target of mir-200c. These results demonstrate that micro-RNAs’ effects on metastasis progression may be different in primary tumor and distant metastases.

## Multiple steps of the metastatic cascade are targeted by oligometastatic miRs

**Fig. 5:**
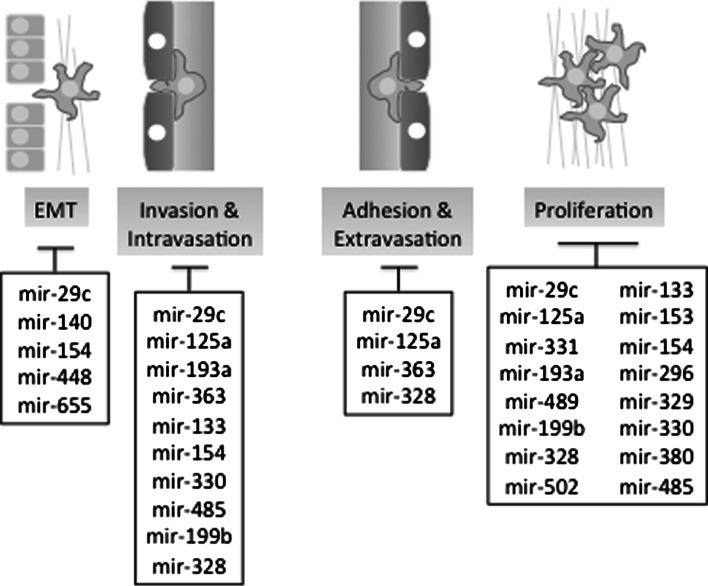
Potential functions of oligomiRs. Multiple micro-RNAs up-regulated in oligometastases datasets (*black* SBRT, *red* Surgery and *blue* both datasets) are each capable of inhibiting multiple points along the metastatic cascade. These include epithelial–mesenchymal transition (EMT); invasion through parenchyma and intravasation into vasculature; adhesion to distant vasculature and extravasation into the distant organ parenchyma; and proliferation in the distal organ. Table 1 details biological processes/functions and down-stream targets of oligomiRs presented in this figure. (Color figure online)

Almost all of the micro-RNAs over-expressed in the surgery oligometastatic samples, and many of those up-regulated in the SBRT oligometastatic samples, have been shown to be tumor-suppressors (Table 1). This suggests that oligometastases development may be associated with miR-based negative regulatory loops which are suppressed in polymetastases evolution. Examination of the molecular pathways predicted to be regulated by oligomiRs from both datasets suggests that they may regulate proliferation, EMT, invasion and motility through multiple down-stream targets (Table 1). Accordingly they may be implicated into the regulation of the distinct metastatic steps (Fig. 5).

Initial formation of pro-metastatic cells predisposed to successful accomplishment of metastatic cascade is commonly associated with epithelial–mesenchymal transition (EMT)— a morphological transformation necessary for escape from the primary tumor and subsequent invasion and metastatic growth. As discussed above, the mir-200c family is a well-known regulator of this mechanism through targeting of the TGF-beta pathway [66–69]. Upstream modulators of ZEB1 and mir-200c expression such as WASF3 and OSM have also been identified to play a role in EMT progression and tumor metastases [70, 71]. Another prominent regulator of EMT is mir-29c, which is up-regulated in oligometastatic patient samples and found to also suppress EMT [72]. Finally, the most highly differentially expressed micro-RNA in the pulmonary oligometastatic samples, mir-655, has recently shown to target ZEB1 and TGFBR2, resulting in similar inhibition of EMT [73]. These data suggest that limited metastatic dissemination seen in oligometastatic patients may partially depend on the control of EMT in the primary tumors (Fig. 5).

Several miRNAs over-expressed in oligometastatic samples have been shown to inhibit tumor cell adhesion and migration. Though these miRNAs have varied targets, their effects converge on mechanisms that are essential to regulation of the cell cytoskeleton and interaction with the extra-cellular matrix. Inhibition at this step can suppress metastasis progression by preventing invasion through the basement membrane in the primary tumor, or adhesion and migration of circulating tumor cells that could otherwise form metastases. Mir-125a is capable of inhibiting Fyn and RhoA, kinases involved in promoting cell adhesion and invasion through cytoskeletal re-arrangements and integrin signaling [74]. Mir-363 has similar effects on neuroblastoma cells and squamous cell carcinomas, inhibiting invasion in vitro and metastasis in vivo partly through suppression of podoplanin, a regulator of adhesion and cytoskeletal structure [75]. Similar findings have been observed in micro-RNAs up regulated in the pulmonary oligometastatic samples. Mir-485 can inhibit breast cancer migration, in addition to inhibition of colony formation in vitro [76]. Mir-296 inhibits Scrib, a regulator of cell motility, thus inhibiting growth in vivo, and repression of this micro-RNA increases tumor invasiveness in vitro [77]. Invasion and adhesion to extracellular matrix is an essential mechanism for metastasis development, and inhibition of this step may result in the low total metastatic burden observed in oligometastases by preventing colonization of distant organs by circulating tumor cells (Fig. 5).

Finally, regulation of proliferation may be critical on the late stages of metastatic cascades, leading to successful colonization (Figs. 2, 5, Table 1). Several micro-RNAs found to be up-regulated in both the SBRT trial and pulmonary resection oligometastases groups are implicated in such regulation. These include, mir-125a & b, mir-29c, mir-133a, mir-193a, mir-331, mir-329 and mir-502 (Table 1) [78–85].The ability of these oligomiRs to inhibit tumor growth is mediated by direct targeting of transcription factors, including the E2F family and cell-checkpoint proteins such as Cyclins D1, D2 and E1 in a variety of carcinoma types. Cyclin pathways, especially Cyclin D1 and its downstream effectors CDK4 and CDK6, represent important targets of tumor-suppressing micro-RNAs. Though the majority of known cell-cycle suppressing micro-RNAs appear to inhibit progression through G1, a few “oncomir” families have been identified that suppress key inhibitors of the cyclin/CDK complexes, p21 and p27 [86, 87]. Tumor cell growth inhibition through cell-cycle arrest may be a significant mechanism of the formation of locally confined slow-growing oligometastases that are amenable to treatment with localized therapy or resection.

**Table 1:** Known functions and down-stream targets of oligomiRs detected in clinical databases of oligometastatic patients

miRNA	Dataset	Oncomir or suppressor	Controlled biological processes	Down-stream targets	References
Mir-655	Surgery	Suppressor	EMT	TGFBR2,ZEB1	[88]
mir-154	Surgery	Suppressor	Proliferation, motility, cell cycle, EMT	TLR2, HMGA2, CCND2	[89–91]
mir-329	Surgery	Suppressor	Proliferation	E2F1	[84]
mir-330	Surgery	Mixed	*Proliferation*, *invasion*, cell cycle, adhesion, apoptosis	SP1, CDC42, SH3GL2	[92, 93]
mir-485	Surgery	Suppressor	Proliferation, migration	MAT1A	[76]
mir-380	Surgery	Oncomir	*Proliferation*, *cell* *cycle*	p53	[94]
mir-298	Surgery	Suppressor	Drug sensitivity	P-gp	[95]
mir-153	Surgery	Mixed	*Proliferation*, apoptosis, drug resistance	PTEN, FOXO3a, FOXO1	[96–98]
mir-296	Surgery	Mixed	Proliferation, *cell cycle*, apoptosis, drug resistance, *angiogenesis*	ICAM1, CDX1, EAG1, Scrib, HMGA1, HGS	[99–102]
mir-448	Surgery	Suppressor	EMT	SATB1	[103]
mir-133a	Surgery	Suppressor	Invasion, proliferation	FSCN1, PNP, TALGN2	[104–106]
mir-328	Both	Suppressor	Invasion, drug resistance, cytokinesis, migration, cell cycle, *proliferation*, ECM degradation	ABCG2, MMP16, KIF23, PTPRJ	[78, 107–110]
mir-502	Both	Suppressor	Proliferation, cell cycle	Rab1B	[85]
miR-199b-5p	Both	Suppressor	Proliferation, drug resistance, invasion	HER2, HES1	[111–113]
miR-125a-3p	SBRT	Suppressor	Invasion, proliferation, apoptosis, cell cycle arrest	FYN, RHOA, BCL2, ALDH1A3, MCLl1, VEGF, MMP11, TNFAIP3	[74, 79, 114–118]
miR-140-5p	SBRT	Suppressor	EMT, proliferation	TGFBR1, FGF9	[119]
miR-29c-3p	SBRT	Suppressor	Proliferation, invasion, adhesion, angiogenesis, drug resistance, apoptosis, cell cycle arrest	MMP2, ITGB1, SIRT1, TIAM1, TNFAIP3, CCNE1, p85, CDC42	[80, 120–124]
miR-489	SBRT	Suppressor	Proliferation	PTPN11	[125]
miR-331-5p	SBRT	Suppressor	Cell cycle	E2F1	[83]
miR-193a-3p	SBRT	Suppressor	Apoptosis, proliferation, EMT, drug resistance, cell cycle	MCL1, EGFR, PLAU, KRAS, p73, E2F6	[82, 126–129]
miR-363-3p	SBRT	Mixed	*EMT*, invasion, proliferation	MBP1, PDPN	[75, 130, 131]

Functions that are up-regulated by a micro-RNA are italicized

## Conclusion

Oligometastasis is a unique phenotype observed in some cases of metastatic cancer. This type of tumor spread is amenable to localized therapy including radiotherapy and resection, resulting in improved outcomes compared to patients with poly-metastatic disease. Analysis of patients’ tumor samples in two different datasets revealed differential micro-RNA expression profiles correlating with metastasis progression, regardless of primary tumor histology. Many micro-RNAs identified in these experiments were up-regulated in oligometastatic lesions and were associated with tumor-suppressive functions. Such “oligomiRs” may be involved in regulation of oligometastatic phenotype during multistep process of metastases development. Biological effects of oligomiRs identified in both datasets substantially overlapped and converged on cell proliferation, migration, adhesion and EMT. These functions are consistent with continuous interactions of the tumor cells with the host microenvironment during dissemination of metastatic cells from the primary tumor to distant sites. The ability to derive microRNA signatures from oligo/polymetastases, combined with an understanding of the pathways regulated by these oligoMirs, may help identify patients with oligometastatic progression with more accuracy than current clinical predictions. An understanding of the biologic basis of oligometastases has the potential to improve patient care through diagnostics and therapeutics. In addition, exploration of the functions of microRNAs linked to oligometastases may give more insight into the underlying mechanisms of metastatic progression and reveal new previously unidentified targets for future therapy.
